# Cytospin-A Regulates Colorectal Cancer Cell Division and Migration by Modulating Stability of Microtubules and Actin Filaments

**DOI:** 10.3390/cancers14081977

**Published:** 2022-04-14

**Authors:** Fan Fan, Jason Roszik, Ling Xia, Susmita Ghosh, Rui Wang, Xiangcang Ye, David Hawke, Lee M. Ellis, Rajat Bhattacharya

**Affiliations:** 1 Department of Colon and Rectal Surgery, The University of Texas MD Anderson Cancer Center, Houston, TX 77030, USA; ifan@mdanderson.org (F.F.); sghosh6@mdanderson.org (S.G.); 2 Department of Melanoma Medical Oncology, The University of Texas MD Anderson Cancer Center, Houston, TX 77030, USA; jroszik@mdanderson.org; 3 Department of Radiation Oncology, The University of Texas MD Anderson Cancer Center, Houston, TX 77030, USA; lxia@mdanderson.org; 4 Department of Surgery, Case Western Reserve University, Cleveland, OH 44106, USA; rxw517@case.edu; 5 Department of Nutrition, Texas A&M University, College Station, TX 77843, USA; xcye@tamu.edu; 6 Department of Systems Biology, The University of Texas MD Anderson Cancer Center, Houston, TX 77030, USA; dhawke@mdanderson.org; 7 Departments of Colon and Rectal Surgery and Molecular and Cellular Oncology, The University of Texas MD Anderson Cancer Center, Houston, TX 77030, USA; lellis@mdanderson.org

**Keywords:** colorectal cancer, cytospin-A, cell division, cell migration

## Abstract

**Simple Summary:**

In this study, we report the effects of depleting cytospin-A (CYTSA), also known as the sperm antigen with calponin homology and coiled-coil domain (SPECC1L) protein, on the proliferation and migration of colorectal cancer (CRC) cells. Mutations in this protein have been previously linked to different developmental disorders. In our studies, depletion of CYTSA in various CRC cells led to significant decreases in proliferation, increases in cell death, and increased formation of multinucleated cells. Knocking down CYTSA also led to severe inhibition of CRC cell migration and invasion. These effects could be related to a significant decrease in the stability of microtubules and alterations in polymerized actin filaments in CYTSA depleted CRC cells. Our studies, for the first time, provide evidence suggesting that targeting CYTSA may be a novel therapeutic strategy for patients with CRC.

**Abstract:**

Proteins that interact with cytoskeletal elements play important roles in cell division and are potentially important targets for therapy in cancer. Cytospin-A (CYTSA), a protein known to interact with actin and microtubules, has been previously described to be important in various developmental disorders, including oblique facial clefting. We hypothesized that CYTSA plays an important role in colorectal cancer (CRC) cell division. The effects of CYTSA depletion on CRC cell proliferation were analyzed using cell growth assays, microscopic analyses of live and fixed cells, and time-lapse imaging. CYTSA depletion led to inhibition of cell proliferation, significant increases in CRC cell death, and accumulation of doublet cells during and following cell division. Depletion of CYTSA also resulted in strong inhibition of CRC cell migration and invasion. Mechanistically, CYTSA depletion resulted in significant decreases in the stability of microtubules and altered polymerization of actin filaments in CRC cells. Finally, bioinformatic analyses were performed to determine the correlation between CYTSA expression and survival of patients with CRC. Interestingly, a strong correlation between high CYTSA expression and poor survival was observed in the TCGA adenocarcinoma data set but not in an independent data set. Since inhibiting CYTSA significantly reduces CRC cell proliferation, migration, and invasion, targeting CYTSA may be a potential novel therapeutic option for patients with metastatic CRC.

## 1. Introduction

Cytoskeletal elements are important for maintaining cell shape and motility, molecular transport, and, most importantly, cell division [1,2,3]. The role of cytoskeletal molecules, especially microtubules and actin, has led investigators to develop therapeutic agents that target cytoskeletal elements for the treatment of various diseases, including cancer [3,4,5]. Various cellular proteins interact with these cytoskeletal factors and influence their function [6,7]. Thus, these cytoskeleton-interacting proteins are also being targeted with the intent to inhibit the function of cytoskeleton structures for therapeutic purposes [8]. Elucidating the biological features of these proteins can not only lead to a better understanding of their role in various diseases but also to the development of better therapeutic agents to inhibit their function.

Cytospin-A (CYTSA), also known as the sperm antigen with calponin homology and coiled-coil domain (SPECC1L) protein, was first identified as an uncharacterized protein that, when mutated, leads to oblique facial clefting, a developmental disorder in humans [9]. Other studies have indicated that mutations in this protein result in Teebi hypertelorism syndrome [10] and, in some cases, autosomal-dominant Opitz G/BBB syndrome [11], a disorder characterized by hypertelorism, hypospadias, other midline defects, and congenital heart defects. In zebrafish, knockdown of a CYTSA homolog resulted in a loss of jaw and facial structures [9,12], and knockdown in Drosophila resulted in phenotypes that were similar to mutations in the integrin signaling pathway that exhibit cell migration defects [9]. Overexpression of GFP-tagged wild-type CYTSA in U2OS cells demonstrates that CYTSA binds to a fraction of microtubules and actin. However, recombinant GFP-CYTSA proteins that harbor mutations found in patients with facial clefting exhibit alterations in microtubule binding properties, suggesting that defects in the cytoskeletal regulatory role of CYTSA may be important in oblique facial clefting [9].

A previous study identified the CYTSA as a substrate of the protein kinase Mps1 and termed it Mps1 interacting protein 1 (Mip1) [13]. Biochemical studies demonstrated that Mip1/CYTSA is a phosphoprotein that interacts with and is phosphorylated by the Mps1 kinase. Knockdown of Mip1/CYTSA in mammalian U2OS cells exhibited chromosome distribution errors that yielded increased numbers of binucleate cells [13].

The role of CYTSA in colorectal cancer (CRC) cells is unknown. We hypothesized that CYTSA is important in CRC cell proliferation and migration. In the current study, we examined the effects of CYTSA depletion in CRC cells and determined whether CYTSA is important in CRC cell proliferation. We also analyzed the importance of CYTSA in CRC cell migration and invasion. Finally, we examined whether the expression of CYTSA is a prognostic factor in patients with CRC.

## 2. Materials and Methods

### 2.1. Cell Culture

All human CRC cell lines were purchased from American Type Culture Collection (ATCC; Manassas, VA, USA). CRC cells were cultured in minimum essential medium (MEM) supplemented with 5% fetal bovine serum (Atlanta Biologicals, Flowery Branch, GA, USA) and the recommended concentrations of vitamins, nonessential amino acids, penicillin or streptomycin, sodium pyruvate, and L-glutamine (Thermo Fisher Scientific, Waltham, MA, USA). All experiments were performed using cells within 15 passages. All cell lines were validated at the MD Anderson Cancer Center characterized cell line core facility.

### 2.2. Reagents

Antibodies for CYTSA (rabbit polyclonal, sc-86401), α-tubulin (mouse monoclonal, sc-23948), acetylated α-tubulin (mouse monoclonal sc-23950), actin (mouse monoclonal, sc-4778), vinculin (mouse monoclonal, sc-25336) and phalloidin conjugated to Alexa-594 (sc-363795) were purchased from Santa Cruz Biotechnology (Dallas, TX, USA). A second anti-CYTSA antibody (rabbit polyclonal, PA5-64448) was purchased from Invitrogen. Anti-pericentrin (rabbit polyclonal, HPA019887) was from Sigma-Aldrich (St. Louis, MO, USA). Goat anti-mouse IgG conjugated to Alexa 594 or goat anti-rabbit IgG conjugated to Alexa 488 were purchased from Thermo Fisher Scientific (Waltham, MA, USA).

### 2.3. Generation of Stable Cell Lines

HCT116 and RKO cells were doubly transfected with the plasmid pCDNA3.1 and a second plasmid that expresses an mCherry-tagged H2B protein [14] and at a ratio of 1:10. Cells were selected in the presence of 250 μg/mL of G418 and then sorted by flow cytometry to identify mCherry-positive cells. These mCherry-H2B cells were then used for further experiments.

### 2.4. siRNA Knockdown

One or two different siRNAs (5′-GAACUGAACAGCCAGGAUA [dT][dT]-3′ and 5′-ACAGAAGGCTATCAGAATA[dT][dT]-3′) that targeted human CYTSA were used to deplete CYTSA in CRC cells. A validated non-targeting siRNA (Sigma-Aldrich, St. Louis, MO, USA) was used as a control [15]. The jetPRIME siRNA transfection reagent (Polyplus, New York, NY, USA) was used according to the manufacturer’s instructions. In brief, the siRNA and jetPRIME reagent were mixed together, incubated for 15 min, and added to CRC cells grown in cell culture medium without antibiotics in six-well plates. After 24 h, cells were washed with phosphate-buffered saline (PBS) and cultured with regular cell culture media with antibiotics. Forty-eight hours from the start of transfection, cells were lysed with either (1) TRIzol^®^ to isolate RNA or (2) radioimmunoprecipitation assay (RIPA) buffer to extract proteins for further analyses.

### 2.5. Cell Growth Assay

In vitro cell growth was assessed using a 3-(4,5-dimethylthiazol-2-yl)-2,5-diphenyltetrazolium bromide (MTT) assay (Sigma) [15]. Briefly, CRC cells were transfected with the required siRNAs for 24 h and then replated into 96-well plates at 3000 cells per well in 200 µL media. The cells were further incubated for 72 h, an MTT assay was then carried out according to the manufacturer’s protocol, and absorption was read at 570 nm.

### 2.6. Colony Formation Assay

CRC cells were transfected with control (Con) siRNA or CYTSA siRNA and replated at ~100 cells/well in 6-well plates and grown for 7–10 days. Colonies were stained with 0.05% methylene blue solution, washed to remove excess dye, and the numbers of colonies were manually counted.

### 2.7. Immunofluorescence Staining

CRC cells were grown on coverslips and fixed with either chilled methanol or formalin for 10 min. Next, slides were treated with PBS containing 1% Tween 20 to permeabilize the cells and washed three times with Tris-buffered saline with 0.05% Tween 20 (TBST). Slides were then blocked in 3% bovine serum albumin in TBST (blocking buffer). After being washed three times in TBST, slides were incubated with primary antibodies that had been diluted in blocking buffer for 1–2 h at ambient temperature. Slides were then incubated with secondary antibodies that were diluted in blocking buffer for 1 h at ambient temperature. Images of stained cells were visualized and captured using an Olympus IX71 microscope and 40× or 60× objectives. DNA was visualized by staining with Hoechst 33,342 (Invitrogen) at 1 μg/mL. Antibodies targeting CYTSA (Invitrogen) α-tubulin (Santa Cruz Biotechnology) were used at 1:100 dilution in the blocking buffer. Stable microtubules were stained using acetyl α-tubulin (Santa Cruz Biotechnology). Actin was stained with phalloidin conjugated to Alexa-594 (Santa Cruz Biotechnology) according to the manufacturer’s instructions. The secondary antibodies used were goat anti-rabbit antibody conjugated to either Alexa-488 or Alexa-594, goat anti-mouse secondary antibody conjugated to either Alexa-488 or Alexa-594 or goat anti-rat secondary antibody conjugated to Alexa-594, as required.

### 2.8. Actin Localization Assessment

CRC cells treated with siRNAs and grown for 48 h were fixed with formalin. Fixed cells were stained with phalloidin conjugated to Alexa-594 and DAPI, and fluorescence images at different focal planes were obtained using a Nikon Eclipse Ti confocal microscope and a 60× objective. Images of cells from a single focal plane at the center of the image stack were analyzed to measure the staining intensity of actin at the cell-cell interphase of post-mitotic doublet cells. Actin intensity at the cell membrane was also determined for the same images. Multiple areas were selected at both positions using the rectangle tool, and intensity was measured using ImageJ. The average actin intensity for cell-cell interphase was divided by the average actin intensity at the membrane of the same cells to determine relative signal intensity.

### 2.9. Live Cell Imaging

HCT116 cells expressing mCherry-H2B were transfected with siRNAs that targeted CYTSA. Twenty-four hours after transfection, the cells were replated and grown overnight in six-well culture plates. Approximately 40 h after transfection, the cells were imaged for 24 h at 15 min intervals in bright-field and with filters to visualize mCherry using a Carl Zeiss Cell Observer microscope and a 20× objective. The microscope was fitted with a chamber to control incubation temperature, humidity, and CO_2_ levels. Cells were grown at 37 °C and 5% CO_2_ during the imaging experiments. Similar experiments were performed with RKO cells.

For analyses, changes in individual cells were followed using both bright-field and fluorescence images. Rounding up of cells and condensation of DNA, as visualized by bright-field and mCherry staining, respectively, were markers of cells initiating mitosis. Normal mitosis was defined when DNA was segregated into two daughter cells, and the shapes of the daughter cells changed from circular to shapes similar to other interphase cells. Cells that showed clear DNA segregation, but the daughter cells failed to separate and stayed as rounded up doublet cells for extended times (>120 min) were counted as cells with defects in daughter cell separation. Cells in which extensive fragmentation of DNA and blebbing of the cell membrane was observed after initiation of mitosis were counted as cells undergoing mitotic death.

### 2.10. Western Blotting

Proteins in cell lysates were separated by sodium dodecyl sulfate polyacrylamide gel electrophoresis following a standard protocol and transferred to Immobilon polyvinylidene membranes (EMD Millipore, Burlington, MA, USA). Membranes were blocked with 5% milk in TBST for 1 h, followed by incubation with primary antibody (diluted in blocking buffer or 2% bovine serum albumin in TBST) overnight. Membranes were then washed three times in TBST, re-incubated with horseradish peroxidase-labeled secondary antibodies for 1 h, washed three times in TBST, and exposed to autoradiography films. Signals were detected by chemiluminescence (Thermo Fisher Scientific). Antibodies for CYTSA, α-tubulin, acetylated α-tubulin, and actin were obtained from Santa Cruz Biotechnology. All antibodies were used according to the manufacturers’ specifications. All cell lysates were prepared in RIPA buffer with protease and phosphatase inhibitors, as described previously [15]. The whole western blot figures can be found in supplementary materials.

### 2.11. MTT and Colony Formation Assays

CRC cells transfected with Con siRNA or CYTSA siRNAs were replated in 96-well plates after 24 h and allowed to grow for a further 72 h. Cell survival was measured by 3-(4,5-dimethylthiazol-2-yl)-2,5-diphenyltetrazolium bromide (MTT) assays, as described previously [16].

For colony formation assays, transfected CRC cells were replated in six-well plates 24 h after being transfected at low densities (150–200 cells/well) and allowed to grow for an additional 7–10 days. The surviving colonies were stained with 0.05% methylene blue solution [17]; imaged and stained colonies were counted using ImageJ software (NIH).

### 2.12. Migration and Invasion Assays

Cell migration in response to increased serum concentrations was assessed using modified Boyden chambers according to the manufacturer’s protocol (Corning, Bedford, MA, USA). Briefly, CRC cells were transfected with control siRNA or CYTSA siRNA. Approximately 48 h after transfection, HCT116 (50,000 cells), SW480 cells (100,000 cells) or HT29 (100,000 cells) in 1% MEM-FBS were incubated on membrane inserts with 8.0-μm pores in 24-well plates. Chemo-attractants (10% MEM-FBS) were placed in the bottom wells. After 24 h (HCT116) or 48 h (other cell lines), cells that did not migrate were removed from the top side of the inserts with a cotton swab. Cells that had migrated to the underside of the inserts were stained with Diff-Quik (Harleco, Gibbstown, NJ, USA) and counted from at least three random fields at 200× magnification using a Nikon Eclipse 55i microscope.

For invasion assays, similar methods were used, except that the cells were placed in the top compartment of modified Boyden chambers with Matrigel-coated membrane inserts (Corning). The numbers of invading cells were quantified from five random fields at 200X magnification.

### 2.13. Bioinformatic Analyses

The Cancer Genome Atlas (TCGA) [18] RNA sequencing and overall survival data were obtained from TCGA repositories. To compare overall survival between groups of high and low gene expression, we divided the patient cohort into two groups: below and above median gene expression. The Kaplan–Meier method was used to analyze survival. We calculated log-rank *p*-values, and differences were considered statistically significant when *p* < 0.05. The integromics data set at MD Anderson Cancer Center with stage I–IV CRC, described earlier [19], was used as a validation data set.

The TCGA data set was also analyzed to compare the expression of CYTSA and various clinical parameters, including tumor stage and microsatellite stability status. Figures were created using the Tableau Desktop software.

The cbioportal (https://www.cbioportal.org, accessed on 28 March 2022) website was used to analyze the TCGA data set to determine mutations in the CYTSA gene among the TCGA data set. The Cancer Cell Line Encyclopedia (Broad, 2019 and Novartis/Broad, 2012) and NCI-60 cell lines data sets were also analyzed for genetic alterations (mutations, amplifications, deletions) in the CYTSA gene.

### 2.14. Statistical Analyses

All graphical calculations and numerical data plotting were performed using Excel (Microsoft, Redmond, WA, USA). Data are expressed as means ± standard deviation. The statistical significance of differences between experimental groups was determined by Student’s *t*-test, and *p*-values < 0.05 were considered significant.

## 3. Results

### 3.1. CYTSA Depletion Inhibits CRC Cell Proliferation

**Figure 1:**
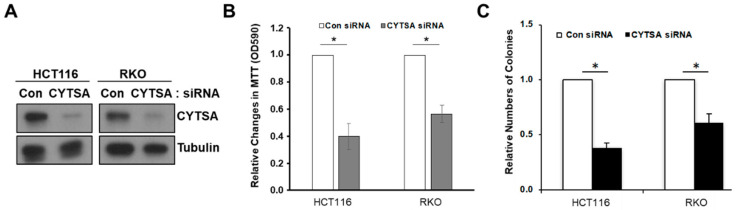
**Depletion of CYTSA in CRC cells inhibit cell proliferation.** (**A**) HCT116 (left panel) or RKO (right panel) cells were treated with Con siRNA or CYTSA siRNA. Levels of CYTSA and tubulin (loading control) from western blots are presented. (**B**) HCT116 and RKO cells transfected with Con siRNA or CYTSA siRNA were grown for 96 h, and cell growth was measured by MTT assay. OD values for Con siRNA were given the value of 1.0, and relative cell growth for CYTSA siRNA-treated cells was plotted. * *p* < 0.05. (**C**) HCT116 or RKO cells treated with Con siRNA or CYTSA siRNA and were grown for 7 days. Numbers of colonies from each set were counted and plotted. Colony numbers of CYTSA siRNA-treated cells are shown relative to those of Con siRNA-treated cells taken as one. The results shown are from at least three experiments. * *p* < 0.05.

To determine whether CYTSA is important in CRC cell proliferation, we first depleted CYTSA in HCT116 and RKO cells using siRNAs (CYTSA siRNA). Depletion was verified at both the RNA (not shown) and protein (Figure 1A) levels using semi-quantitative RT-PCR and Western blotting, respectively. To determine whether depletion of CYTSA in CRC cells affected CRC cell proliferation and survival, we performed growth assays with CRC cells treated with Con siRNA and CYTSA-targeting siRNAs. MTT assays performed 72 h after siRNA transfection demonstrated a strong decrease in cell proliferation when CYTSA was depleted in HCT116 and RKO cells (Figure 1B). Colony formation assays also demonstrated that CYTSA depletion using siRNAs significantly reduced the number of viable CRC colonies (Figure 1C and Supplementary Figure S1). Similar results were obtained with SW480 and HT29 cell lines (Supplementary Figure S1).

### 3.2. Depletion of CYTSA Lead to Defects in CRC Cell Division and Increased Cell Death

**Figure 2:**
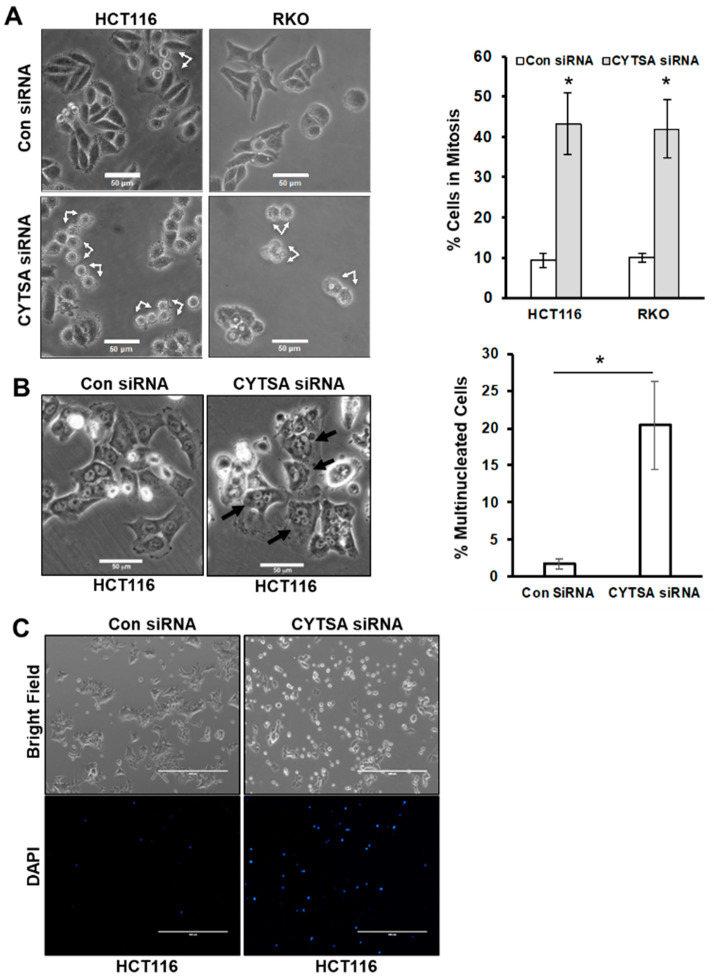
**Depletion of CYTSA results in cell division defects in CRC.** (**A**) Bright-field images are shown of HCT116 or RKO cells treated with Con siRNA and CYTSA siRNA at 48 h after siRNA transfection (left panel). Post-mitotic doublet cells are indicated by arrows. Scale bar = 50 μm. Images from Con siRNA or CYTSA siRNA transfected sets were manually counted to determine the percentage of mitotic or post-mitotic doublet cells. Results are plotted for HCT116 and RKO cells from at least three experimental sets (right panel). * *p*-value < 0.05. (**B**) Bright-field images of HCT116 cells were obtained 4 days after being transfected with Con siRNA or CYTSA siRNA (left panels). Multinucleated cells are designated with black arrows for the CYTSA siRNA set. Scale bar = 50 μm. (**C**) HCT116 cells were transfected with either Con siRNA (left panels) or CYTSA siRNA (right panels). Cells were grown for 72 h; DAPI was added to the media, and images were taken with a 4× objective. Bright-field (top panels) or fluorescence images for DAPI (lower panels) are shown. CYTSA siRNA-treated cells have a significantly higher number of dead and dying cells that take up DAPI than do Con siRNA-treated cells.

To determine the effects of CYTSA depletion on CRC cell growth, we analyzed CRC cell lines with KRAS mutations (HCT116, SW480) and BRAF mutations (RKO, HT29). Most studies were performed in HCT116 and RKO cell lines, and some key experiments were validated in SW480 and HT29 cells. We transfected HCT116 and RKO cells with either control or CYTSA siRNAs. Microscopic analyses of CRC cells after 2 days following transfection with CYTSA siRNA demonstrated a very significant accumulation of mitotic cells (~40–50%) (Figure 2A). Interestingly, a large proportion of these were doublet cells that were similar to cells in the late stages of cell division. CRC cells treated with a non-targeting control siRNA (Con siRNA) [15] demonstrated the expected normal proportion of mitotic cells (<10%) progressing through normal cell division (Figure 2A). Similar increases in the post-mitotic doublet cells were also observed for SW480 and HT29 cells (Supplementary Figure S2). Additionally, analyses of the transfected cells after 4 days demonstrated that the CYTSA siRNA-transfected cells had a significant accumulation of large multinucleated cells (Figure 2B), indicative of mitotic defects. In contrast, the Con siRNA-treated HCT116 cells had very few multinucleated cells that usually arise spontaneously in any HCT116 cell population.

To determine if CYTSA depletion also induced cell death, the live cell impermeable DNA staining dye DAPI was added to the culture media at 3 days post siRNA transfection. Imaging of the plates using bright-field and fluorescent microscopy indicated that very few Con siRNA-treated cells took up DAPI (Figure 2C). However, there was a large increase in DAPI-stained cells in the CYTSA siRNA-transfected set (Figure 2C); this staining marked dead cells and cells with compromised membrane integrity, an indicator of impending cell death.

### 3.3. Depletion of CYTSA Enhances Mitotic Cell Death and Post-Mitotic CRC Cell Segregation

**Figure 3:**
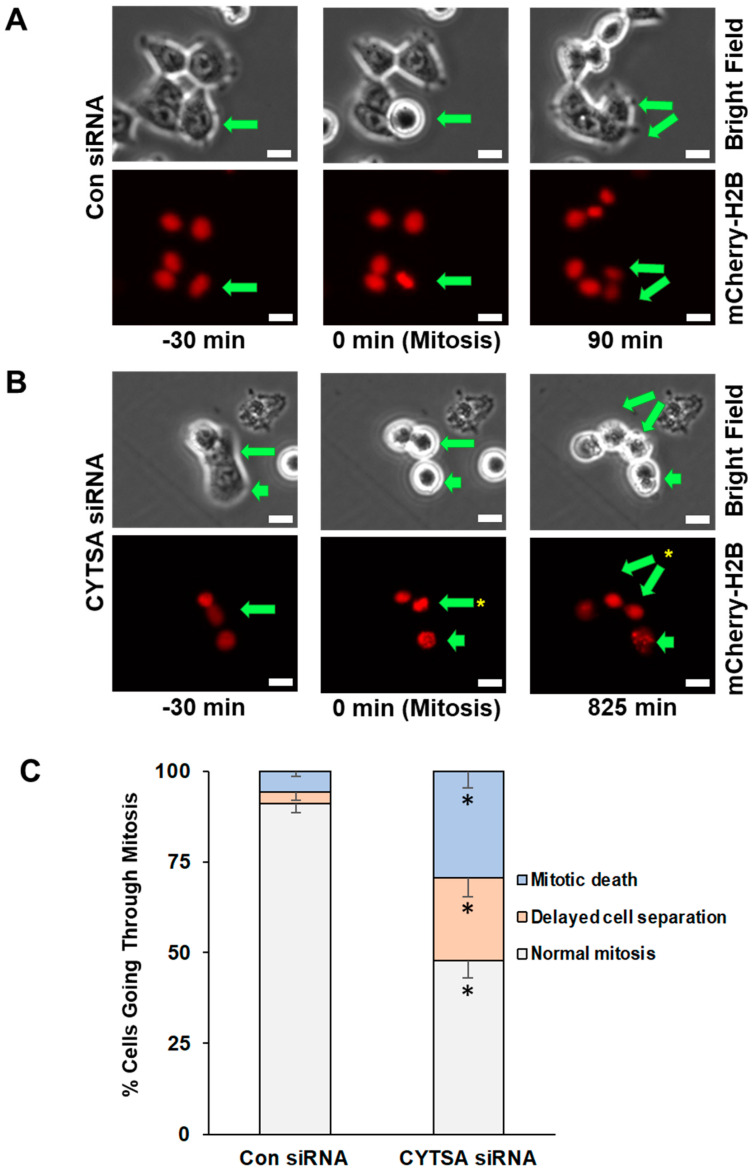
**Depletion of CYTSA enhances mitotic cell death and post-mitotic CRC cell segregation.** HCT116 cells that stably expressed mCherry-H2B were transfected with Con siRNA or CYTSA siRNA and grown for 40 h; real-time imaging was performed for the next ~24 h at 15 min intervals. Bright-field (top) and fluorescence images (mCherry-H2B; bottom) were obtained. (**A**) Sequence of images of Con siRNA-treated cells are shown. A CRC cell that goes through mitosis is indicated by an arrow. The cell initially in interphase (left panel) enters mitosis as determined by condensation of the DNA or circular phenotype (middle panels) and completes cell division (daughter cells marked by arrows, right panel). Time (in minutes) at which the images were obtained is shown below the images. Scale bar = 20 μm. (**B**) Sequence of images of CYTSA siRNA-treated cells are shown. Mitotic cells, as determined on the basis of metaphase DNA or a circular phenotype, are marked by arrows (middle panels). Non-separated daughter cells (marked by two arrows) are shown (right panels). A different mitotic cell (short arrow) that undergoes DNA fragmentation and cell membrane blebbing is also shown (right panel). Time (in minutes) at which the images were obtained is shown below the images. Scale bar = 20 μm. (**C**) Percentage of mitotic cells going through; (i) normal mitosis, (ii) showing delay in daughter cell separation, or (iii) with evidence of mitotic death were plotted for CRC cells transfected with Con siRNA or CYTSA siRNA. Note: Significant differences were found for similar outcomes (e.g., normal mitosis) when compared between Con siRNA vs. CYTSA siRNA-treated cells. * *p* < 0.05.

To determine if CYTSA depleted CRC cells were undergoing cell death following mitosis, we performed time-lapse microscopy of live HCT116 and RKO cells that expressed mCherry-H2B and had been transfected with Con siRNA or CYTSA siRNA. After transfection, the cells were allowed to grow for ~40 h to deplete CYTSA. Cells were imaged for ~24 h, and individual cells were followed from the start to the end of cell division. Bright-field images were used to determine cell shape, and mCherry-H2B images were used to visualize DNA segregation. An analysis of Con siRNA-transfected HCT116 cells (*N* = 140) indicated that most cells progressed through cell division normally (91%), while a small number of cells either had a delay in the separation of daughter cells following mitosis (~3%) or had catastrophic cell death (~5%) following mitosis failure (Figure 3A,C). In contrast, CYTSA siRNA-treated cells (*N* = 116) had a significantly reduced number of cells undergoing normal cell division (~48%) and a significant increase in cells that completed DNA segregation but were stuck as doublet cells for extended periods of time (~23%) or exhibited catastrophic cell death (~29%) following mitosis (Figure 3B,C). Similar observations were also noted in RKO cells (Supplementary Figure S3).

### 3.4. CYTSA Regulates CRC Cell Migration and Invasion

**Figure 4:**
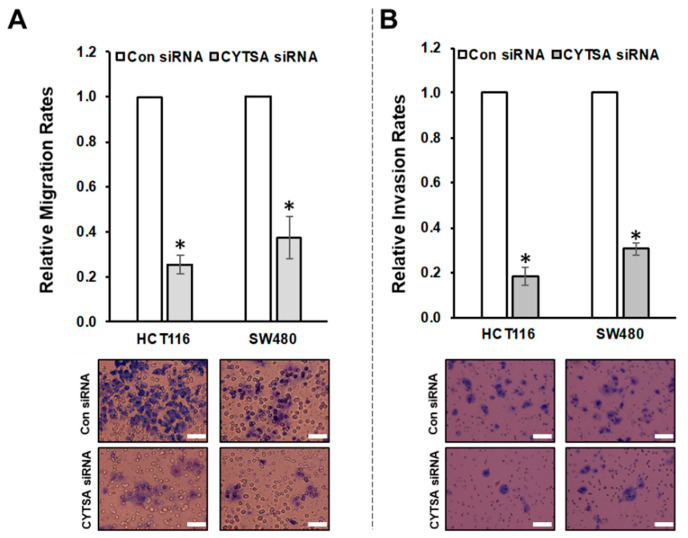
**CYTSA regulates migration and invasion of CRC cells.** (**A**) HCT116 and SW480 cells were transfected with Con siRNAs or CYTSA siRNAs, and migration of these cells were measured using Boyden chambers. Relative migration rates are plotted with migration rate for Con siRNA-treated cells taken as 1.0. Representative images of the membranes are shown below. * *p*-value < 0.05. (**B**) Relative invasion rates of HCT116 and SW480 cells transfected with Con siRNA or CYTSA siRNA are plotted. Representative images of the membranes from the invasion assays are shown below. * *p*-value < 0.05. Scale bar = 50 μm.

To determine if CYTSA had any effect on CRC cell migration, we examined the effects of depleting CYTSA in HCT116 and SW480 cells that we have previously shown can be reliably used to measure cell migration [15]. Migration of these cells transfected with Con or CYTSA siRNA was measured using Boyden chambers. CRC cells depleted of CYTSA exhibited significantly reduced cell migration (Figure 4A) compared to control cells. Similar effects were observed for HT29 cells (Supplementary Figure S4). Effects of CYTSA depletion were also assessed on HCT116 cells using scratch assays. CYTSA siRNA-treated HCT116 cells showed a strong reduction in cell migration as compared to cells treated with control siRNAs (Supplementary Figure S4).

Next, we used HCT116 and SW480 cells to determine the effects of CYTSA depletion on invasion in response to stimuli (high serum). HCT116 and SW480 cells transfected with CYTSA siRNA invaded the membranes at significantly lower rates than did cells treated with control siRNA (Figure 4B). (Note: HT29 cells failed to invade through the Matrigel-coated membranes within our experimental time period of 48 h and thus could not be used for the study.)

### 3.5. Depletion of CYTSA Reduces Stability of Microtubules in CRC Cells

**Figure 5:**
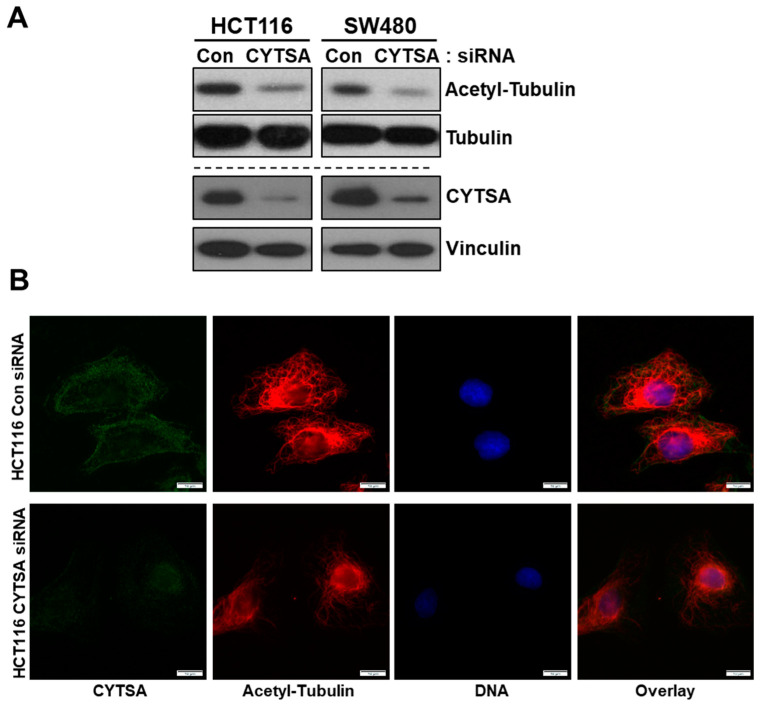
**CYTSA depletion reduces the stability of microtubules in CRC cells.** (**A**) Lysates of HCT116 and SW480 cells transfected with Con siRNA and CYTSA siRNA were analyzed for levels of acetylated α-tubulin and tubulin by Western blotting. The same lysates were also analyzed for levels of CYTSA to validate the knockdown of CYTSA. Vinculin was measured as a loading control. (**B**) HCT116 cells transfected with either Con or CYTSA siRNA were fixed in methanol and stained with antibodies against CYTSA (green) and acetylated α-tubulin (red). Nuclei were stained with Hoescht. Scale bar = 10 μm.

Previous studies indicated that CYTSA may affect the stability of microtubules and actin [9]. We examined the effects of CYTSA depletion on microtubule stability in CRC cells. Depletion of CYTSA in HCT116 and SW480 led to a significant decrease in the levels of stable microtubules as measured by acetylated α-tubulin content in these cells and compared to cells treated with Con siRNA (Figure 5A). Similar changes were observed in other CRC cell lines (Supplementary Figure S5A). The observations derived from western blots were validated by immunostaining of CRC cells. CRC cells depleted in CYTSA demonstrated significantly reduced acetylated α-tubulin staining as compared to cells with normal CYTSA levels (Figure 5B and Supplementary Figure S5B).

### 3.6. Lack of CYTSA Affects Actin Polymerization in CRC Cells

**Figure 6:**
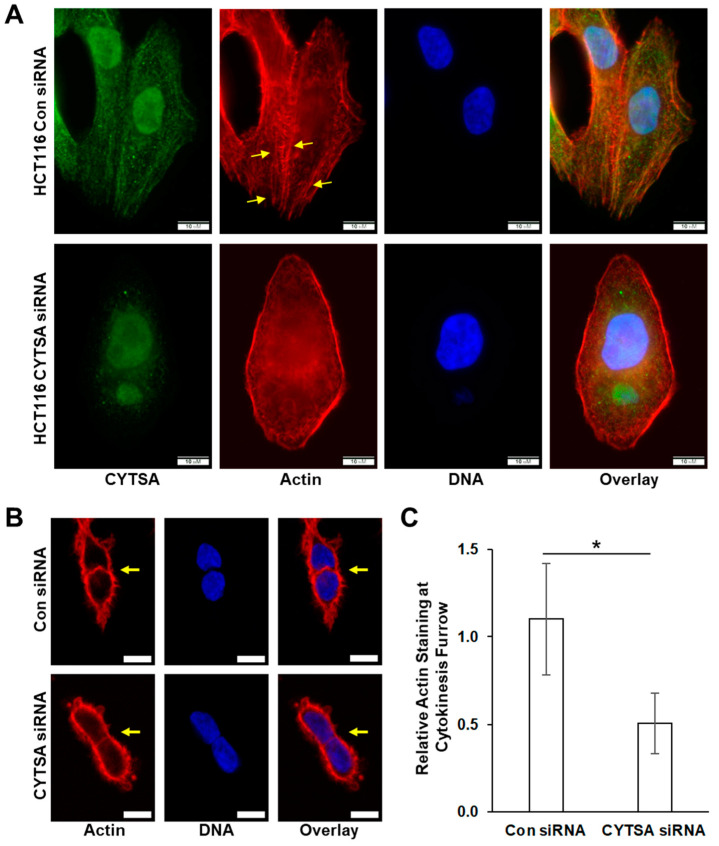
**Depletion of CYTSA alters actin polymerization in HCT116 cells.** HCT116 cells were transfected with either Con siRNA or CYTSA siRNA and grown for 48 h. (**A**) Representative HCT116 that have been stained for CYTSA (green), actin (red), and DNA (blue) and treated with either Con siRNA (top panels) or CYTSA siRNA (bottom panels) are shown. Note: formalin-fixed HCT116 cells demonstrate non-specific nuclear staining with the CYTSA antibody. Scale bar = 10 μm. (**B**) Single planes of Z-stack images of HCT116 cells stained for actin (red) and DNA (blue) are shown. Con siRNA (top panels)- and CYTSA siRNA (bottom panels)-treated cells are shown. Note that the actin staining at the cell-cell interphase (pointed by arrows) of cells undergoing cytokinesis was significantly weaker in CYTSA siRNA-treated cells than those in the Con siRNA-treated cells. Scale bar = 10 μm. (**C**) Bar plots showing actin staining at cytokinetic furrow relative to actin at the cell periphery. * *p*-value < 0.05.

We analyzed actin organization in CRC cells, with or without CYTSA expression. CYTSA depletion in interphase HCT116 cells resulted in fewer long cytoplasmic actin filaments as compared to HCT116 cells with normal CYTSA levels (Figure 6A), with some cells exhibiting mostly peripheral actin bundles with a significant loss in cytoplasmic actin filaments (Figure 6A). Similar results were observed in SW480 cells (Supplementary Figure S6). Further, analyses of doublet cells indicated that cells lacking CYTSA had significantly weaker staining for polymerized actin at the cytokinetic furrow as compared to similar cells with normal CYTSA protein expression (Figure 6B). Using actin levels at the cell periphery as an internal control, we measured the relative actin signal at the cell-cell junction in post-mitotic doublet cells. Polymerized actin at the cell-cell junction was significantly lower in CYTSA siRNA-treated cells as compared to cells treated with control siRNA (Figure 6C). Together, these data indicate that CRC cells without CYTSA protein have significant defects in actin organization and stability.

### 3.7. CYTSA Expression Is Likely Related to Poorer Outcomes in Patients with mCRC

**Figure 7:**
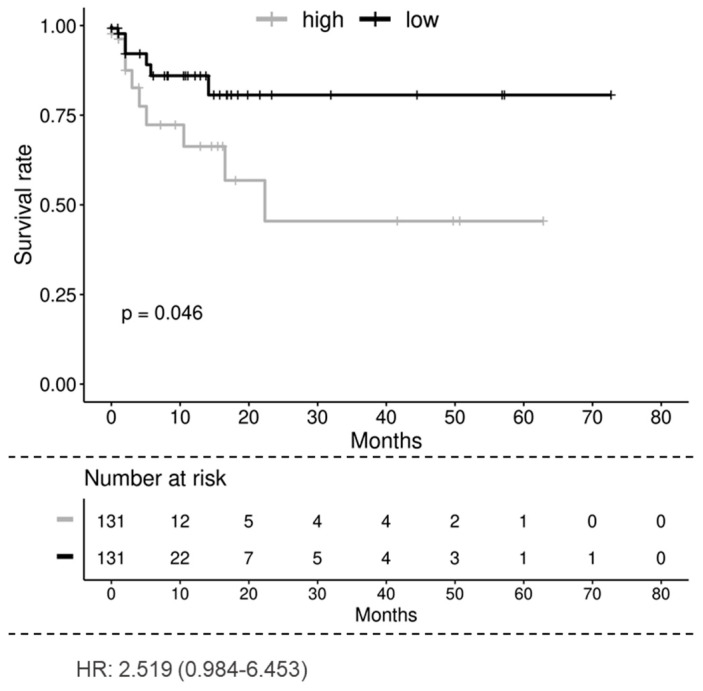
**Overall survival of patients with low and high CYTSA expression.** Kaplan–Meier analysis of the TCGA colorectal adenocarcinoma data set shows that the low CYTSA gene expression group (below median expression, *N* = 131, black curve) is associated with significantly better survival than the high CYTSA expression group (above median expression, *N* = 131, gray curve). *p* < 0.05. Numbers of patients at risk at various time points and the hazard ratio are shown.

To determine if CYTSA expression is related to CRC disease progression and outcomes, an analysis of the TCGA colorectal adenocarcinoma data was performed. Analyses of the TCGA data set where patients were separated into groups that had higher or lower than a median expression of CYTSA indicated a strong correlation between high CYTSA expression and worse clinical outcomes (*p* < 0.05) (Figure 7). However, analyses of an independent data set at MD Anderson Cancer Center with stage I–IV CRC samples [19] did not demonstrate a significant correlation between high CYTSA expression and poor clinical outcomes (Supplementary Figure S7A). Therefore, we note that we have not been able to validate this association with overall survival. In addition, analyses of the TCGA data set showed no significant differences in CYTSA expression among CRC tumors with different MSI statuses and tumor stages (Supplementary Figure S7B,C).

## 4. Discussion

We demonstrated that CYTSA plays a critical role in the proliferation of CRC cells. Depletion of this protein leads to increases in cell death and the formation of multinucleated cells, both resulting from defects in normal cell division. We also demonstrate that depletion of CYTSA strongly inhibits CRC cell migration and invasion. Our studies indicate that depletion of CYTSA from CRC cells leads to a significant decrease in the stability of microtubules. Loss of CYTSA also leads to alterations in actin organization in both interphase and dividing CRC cells. While CYTSA depleted CRC cells at interphase had fewer long actin filaments within the cell body, polymerized actin levels are significantly reduced at the cell-cell interphase of post-mitotic cells. As actin is a key element in the segregation of daughter cells through cytokinesis after successful mitosis, defects in actin function explain the large abundance of post-mitotic doublet cells and the large increase in multinucleated cells after CYTSA depletion; both markers of cell segregation defects. Additionally, our analyses of the TCGA database suggest that higher expression of CYTSA is related to poorer survival in patients with CRC. Thus, with CYTSA affecting both CRC cell proliferation and migration, we propose that targeting CYTSA may be a novel therapeutic strategy for study in patients with mCRC.

CYTSA has been previously shown to be important in various developmental disorders [9,10,11,20], and distinct mutations in CYTSA have been found to be correlated with these diseases. These mutations have been evaluated using GFP-CYTSA overexpression in U2OS cells and have been found to lead to altered binding of the protein to microtubules and actin [9]. Such alterations lead to cell adhesion and migration defects and likely result in facial morphogenesis defects [9]. However, the effects of such CYTSA mutations on the division of mesenchymal cells participating in these developmental processes are unknown. A more recent study proposed a role for CYTSA in regulating PI3K-AKT in addition to its role in modulating the adherens junction [21]. However, the importance of CYTSA in the proliferation and migration of cancer cells, especially CRC cells, is not well understood. Our studies unequivocally demonstrate that CYTSA, in addition to mediating important roles in developmental processes, is also critical for the proliferation and migration of CRC cells.

Various CYTSA mutations lead to various developmental disorders. However, the effects of such mutations on CRC cell proliferation are currently unknown. Analyses of the TCGA data set using the cbioportal website (https://www.cbioportal.org, accessed on 28 March 2022) have identified only a few mutations in the CYTSA gene in patients with CRC; six missense mutations and one mutation leading to a truncated protein. Although the effects of these mutations on the progression of CRC are yet unknown, the N1041D, A1016T, and G1083D mutations are all located in the calponin homology domain (CHD) and thus can likely affect actin and tubulin binding [11,13] and influence proliferation and migration of CRC cells. Mutations in G1083 have been observed in both patients with the Opitz G/BBB syndrome [11] and CRC. Expression of recombinant CYTSA with mutated G1083 in U2OS cells results in reduced stable microtubules [11], suggesting that this mutation affects CYTSA function. A recent study suggested a role for the coiled-coil domain 2 (CCD2) of CYTSA in associating with microtubules and deletion of the CCD2 results in gain-of-function mutations [22]. However, none of the mutations in CYTSA found in patients with CRC are located in the CCD2 domain and thus likely to act as gain-of-function mutations. Future studies on expressing mutated CYTSA proteins will reveal if these mutations have functional significance in CRC cells.

Although mutations in CYTSA have been implicated in various developmental disorders, our study is the first to examine the correlation between CYTSA expression and outcomes in patients with CRC. Our studies demonstrating that CYTSA is critical for CRC cell proliferation support the hypothesis that CRC cells with very low CYTSA may have defects in tumor cell proliferation and thus lead to a better prognosis. Conversely, as CRC cells lacking CYTSA have lower migration and invasion rates, it is possible that patients with higher CYTSA have increased rates of metastasis and, in turn, lead to a poorer prognosis. Our analyses of the RNA expression from the TCGA adenocarcinoma database indicated that patients with higher than median CYTSA expression had poorer survival than patients with lower than median CYTSA expression (Figure 7). However, our secondary analyses of an independent data set failed to show a strong correlation between CYTSA expression and patient survival (Supplementary Figure S7). Thus, our studies do not unambiguously demonstrate that CYTSA expression can be correlated to the survival of patients with CRC. Analyses of the TCGA data set also do not show any significant differences in CYTSA expression and CRC tumor stage or MSI/MSS status (Supplementary Figure S7). One of the drawbacks of our study is the relatively small number of samples in the data sets analyzed. Further studies determining levels of CYTSA proteins in independent larger cohorts of CRC tissue microarrays with both primary and metastatic tumor samples and correlating CYTSA protein levels to patient survival and other clinical parameters will provide a better understanding of the effects of CYTSA alterations on CRC tumor growth and metastasis.

Recent studies have discovered the presence of various CYTSA-fusion proteins in various cancers. CYTSA-RET fusions in spindle cell tumors [23] and uterine cervical sarcoma [24], CYTSA-NTRK fusions in pediatric sarcoma and brain tumors [25], and mesenchymal tumors of the gastrointestinal tracts [26] have been described. Interestingly, CYTS-ALK [27] and CYTSA-MET [28] fusions have been identified in patients treated with ALK or EGFR inhibitors, suggesting that these fusion proteins may drive therapy resistance. How regions of CYTSA fusing with these various kinases render them hyper-active is yet unknown. It is possible that such fusion events lead to autophosphorylation and activation of the kinase domains. Although our studies show the full-length CYTSA to be important in cell proliferation and migration, a better understanding of the functional aspects of the various domains of CYTSA may provide insights into the activities of these fusion proteins.

A previous study in U2OS cells examined CYTSA (it was not yet named CYTSA) as a substrate of the Mps1 kinase and named the protein Mps1 kinase-interacting protein 1 (Mip1) [13]. This study indicated that depletion of CYTSA could lead to aberrant actin organization and spindle misfunction during early cytokinesis and resulted in an increase in bi-nucleated cells. In our studies, we demonstrate that apart from increases in cell death following initiation of mitosis, depletion of CYTSA also affects a significant fraction of CRC cells during late stages of cytokinesis that result in impaired separation of daughter cells. This is likely due to problems at the cytokinetic furrow resulting from reduced polymerized actin at that location. Although our present study and previous studies [9,13] have revealed that CYTSA depletion affects the function of actin and microtubules, the resulting effects may be manifested differently in different cell types.

One of the major findings of our study is that depletion of CYTSA leads to a decrease in microtubule stability (Figure 5). Previous studies have shown that when CYTSA is overexpressed in cells, recombinant CYTSA co-localizes with stable microtubules [9,11]. Cells expressing specific CYTSA mutants that are important in developmental disorders appeared to have lower acetylated-tubulin, a marker for stable microtubules [9,11]. However, no direct evidence that wild-type CYTSA can regulate the stability of microtubules has been reported. Our studies, both through Western blotting of whole-cell lysates and fluorescence imaging of individual CRC cells, demonstrate that removal of CYTSA from CRC cells reduces stable microtubule content (Figure 5). These findings indicate that CYTSA may function as a stabilizer of microtubules. Various proteins have been identified that bind to microtubules. While some such proteins bind to microtubules and help the movement of various cellular components along microtubule tracks (e.g., myosin, kinesins, and dynein [29]), others can bind to microtubules and either stabilize (e.g., Tau, Map2 [30]) or destabilize the microtubules (e.g., MCAK [31], Stathmin [32]). However, the exact mechanism by which CYTSA regulates microtubule stability is yet to be determined.

Our findings also indicate that CYTSA can regulate the stability and likely function of actin filaments (Figure 6). CYTSA has been shown to regulate actin structure in mitotic [13] or interphase cells [9]. Our studies not only show alterations in actin assembly in interphase cells lacking CYTSA (Figure 6), we additionally show that actin organization is affected at the cytokinetic furrow (Figure 6). These findings are consistent with reports that actin and CYTSA co-localize at the cytokinetic furrow [13] and indicate that presence of CYTSA is important for normal actin polymerization at the furrow. Different functions of actin are regulated by multiple actin-binding proteins, and many such proteins regulate actin function during cytokinesis [33]. Various proteins, including anillin [34], RhoA [35], or the ezrin, radixin, and moesin (ERM) family of proteins [36], play important roles in the formation and function of the cytokinetic furrow and ring formation by regulating the recruitment and stabilization of actin. Although our data indicate that CYTSA regulates actin polymerization in both interphase and dividing cells, it is still unknown if these effects are mediated through its direct interactions with actin or by perturbing the function of other actin-binding proteins. Further studies on the molecular mechanisms of CYTSA function will likely provide insights into its role in the regulation of actin.

In our studies, we also examined 14 different CRC cell lines, including cells that had defects in DNA mismatch repair or a high microsatellite instability phenotype, and determined that all had CYTSA protein expression (Supplementary Figure S8A). Cells with such genetic defects that have been propagated over long periods of time tend to lose different genes; however, only essential genes, including genes that are important in cell division, are usually preserved [37]. Retaining the expression of CYTSA in all cell lines strongly suggests that it is one of the essential genes required for cell proliferation. In addition, analyses of publicly available gene expression data from approximately 2800 different cancer cell lines indicated that very few cell lines lack CYTSA expression or have mutations that may possibly affect its function (Supplementary Figure S8B). Together, these findings strongly suggest that CYTSA plays an essential role in the proliferation of not only CRC but almost all cancer cell types.

## 5. Conclusions

Multiple cytoskeleton-interacting proteins have been extensively studied to determine their role in regulating cancer cell division and target these proteins for cancer therapy. The results of our studies indicate that CYTSA is critical for the division and migration of CRC cells, and knocking down this protein leads to strong inhibition of both CRC cell proliferation and migration. Thus, CYTSA should be considered a novel target for the development of anti-cancer therapeutics. Future studies on elucidating the detailed mechanism of CYTSA function will not only provide better insights into the roles of this protein but will also help identify possible therapeutic agents to target this protein and/or its binding partners for cancer therapy.

## Data Availability

All data to support the findings and conclusions of this study are included in the article.

## Supplementary Materials

The following supporting information can be downloaded at: https://www.mdpi.com/article/10.3390/cancers14081977/s1, Supplementary materials: the western blot figures, Figure S1: Effect of CYTSA depletion on CRC colony formation, Figure S2: Depletion of CYTSA results in cell division defects in CRC, Figure S3: Depletion of CYTSA enhances mitotic cell death and post-mitotic segregation in RKO cells, Figure S4: CYTSA regulates migration and invasion of CRC cells, Figure S5: CYTSA depletion reduces stability of microtubules in multiple CRC cells, Figure S6: Depletion of CYTSA alters actin polymerization in SW480 cells, Figure S7: Comparison of CYTSA expression levels and patient survival, and CRC tumors with different microsatellite instability status and age, Figure S8: CYTSA is expressed in almost all cancer cell lines.
