# Supplementary material for: Cytospin-A Regulates Colorectal Cancer Cell Division and Migration by Modulating Stability of Microtubules and Actin Filaments
Source: Cancers (Basel). 2022 Apr 14;14(8):1977. doi: 10.3390/cancers14081977 (PMC9026928; doi:10.3390/cancers14081977)
Supplement: Supplementary file 1 [file cancers-14-01977-s001.zip › cancers-1646065-SM.pdf]

cancers

Cytospin-A Regulates Colorectal Cancer Cell Division and Migration by Modulating Stability of Microtubules and Actin Filaments

Fan Fan [1], Jason Roszik [2], Ling Xia [3], Susmita Ghosh [1], Rui Wang [4], Xiangcang Ye [5], David Hawke [6], Lee M. Ellis [7] and Rajat Bhattacharya [1,*]

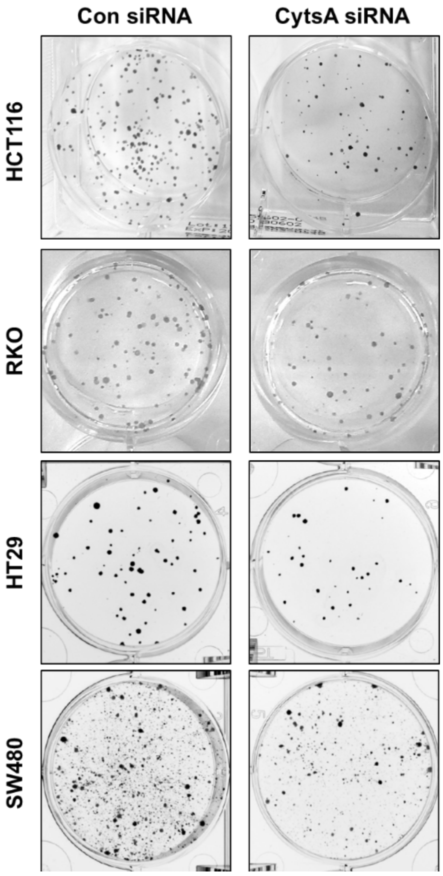

Supplementary Figure S1: Effect of cytospin-A (CYTSA) depletion on CRC Colony Formation. Different CRC cells were transfected with Con siRNA or CYTSA siRNA and equal numbers of cells (~100-200) from each set were plated for each cell line. Colonies were allowed to form, stained with 0.5% methylene blue solution and images were obtained.

Cancers **2022**, *14*, 1977. https://doi.org/10.3390/cancers14081977 www.mdpi.com/journal/cancers

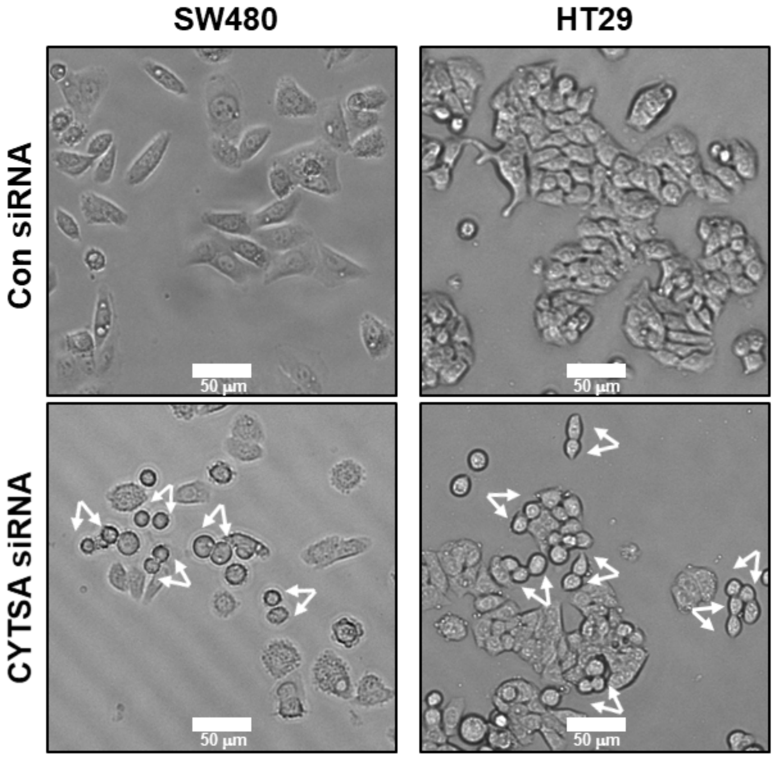

Supplementary Figure S2: Depletion of CYTSA results in cell division defects in CRC. Bright-field images are shown of SW480 or HT29 cells treated with Con siRNA and CYTSA siRNA at 48 hours after siRNA transfection. Post-mitotic doublet cells are indicated by arrows. Scale bar = 50 μm.

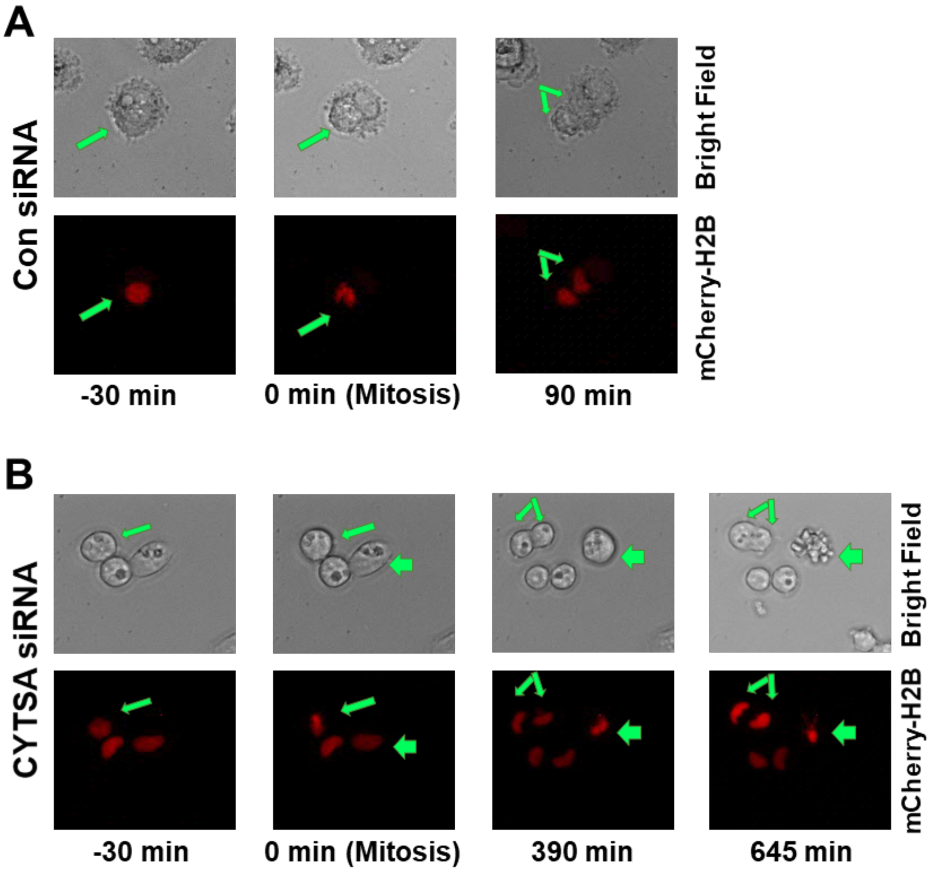

Supplementary Figure S3: Depletion of CYTSA enhances mitotic cell death and post-mitotic segregation in RKO cells. RKO cells expressing mCherry-H2B were transfected with Con siRNA or CYTSA siRNA and real-time imaging was performed for the next ~24 hours at 15 min intervals. Bright-field (top) and fluorescence images (mCherry-H2B; bottom) were obtained. **A)** Sequence of images of Con siRNA-treated cells is shown. A CRC cell that goes through mitosis is indicated by an arrow. Time at which images were obtained is shown below the images. **B)** Sequence of images of CYTSA siRNA-treated cells are shown. Mitotic cells, as determined on the basis of metaphase DNA or a circular phenotype, are marked by arrows (middle two panels). Non-separated daughter cells (marked by two arrows) are shown (right panels). A different mitotic cell (short arrow) that undergoes DNA fragmentation and cell membrane blebbing is also shown. The time at which the images were obtained are shown below.

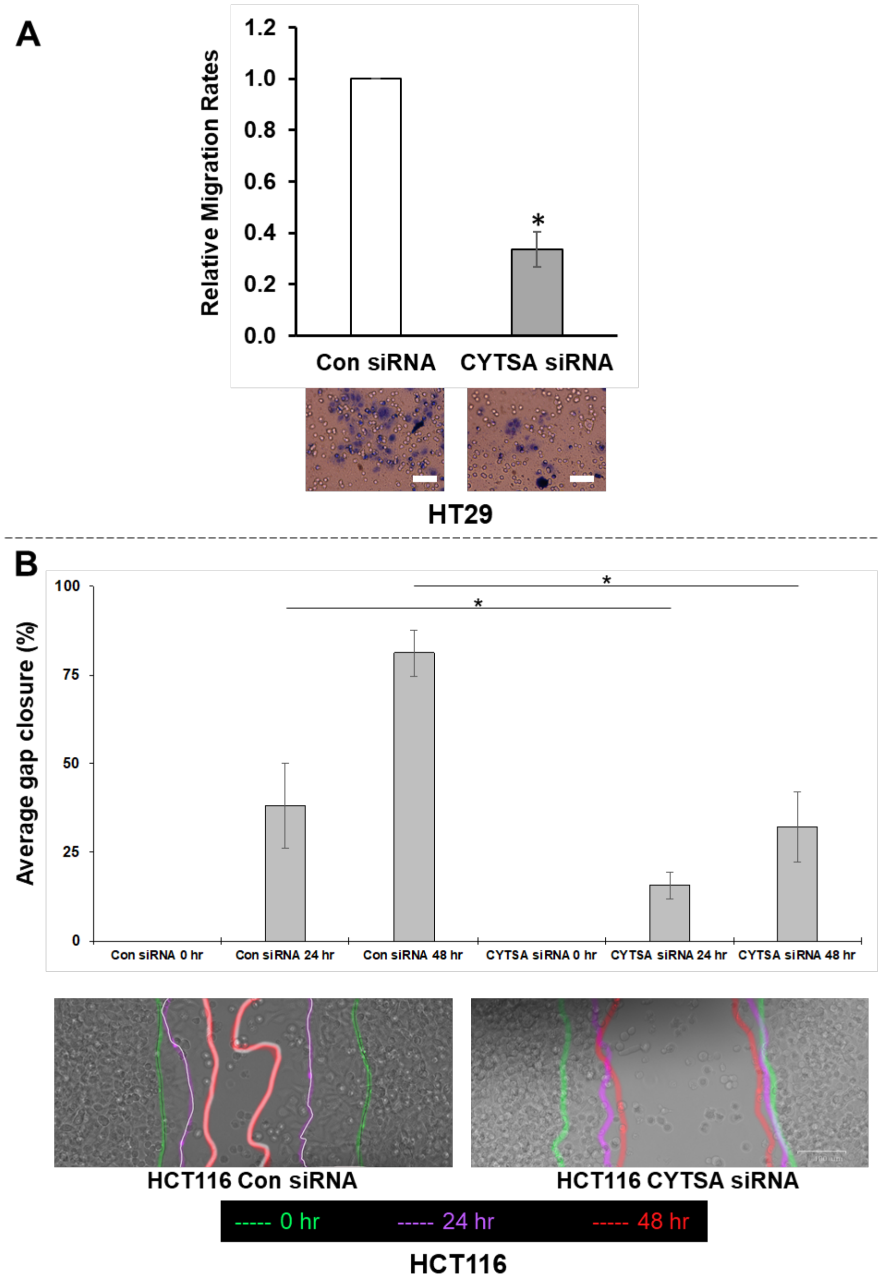

Supplementary Figure S4: CYTSA regulates migration and invasion of CRC cells. A) HT29 cells were transfected with Con siRNAs or CYTSA siRNAs and migration of these cells were measured using Boyden chambers. Relative migration rates are plotted with migration rate for Con siRNA treated cells taken as 1.0. Representative images of the membranes are shown below. * *P* value < 0.05. Scale bar = 50 µm. B) Scratch assay of HCT116 cells demonstrates reduced migration in absence of CYTSA. Scratch assays were performed using HCT116 cells transfected with Con or CYTSA siRNA. Average gap closures were plotted at 0, 24 and 48 hours. Representative composite images of the plates are shown below. Position of scratch boundaries at various time points are indicated on the images. * *P* < 0.05.

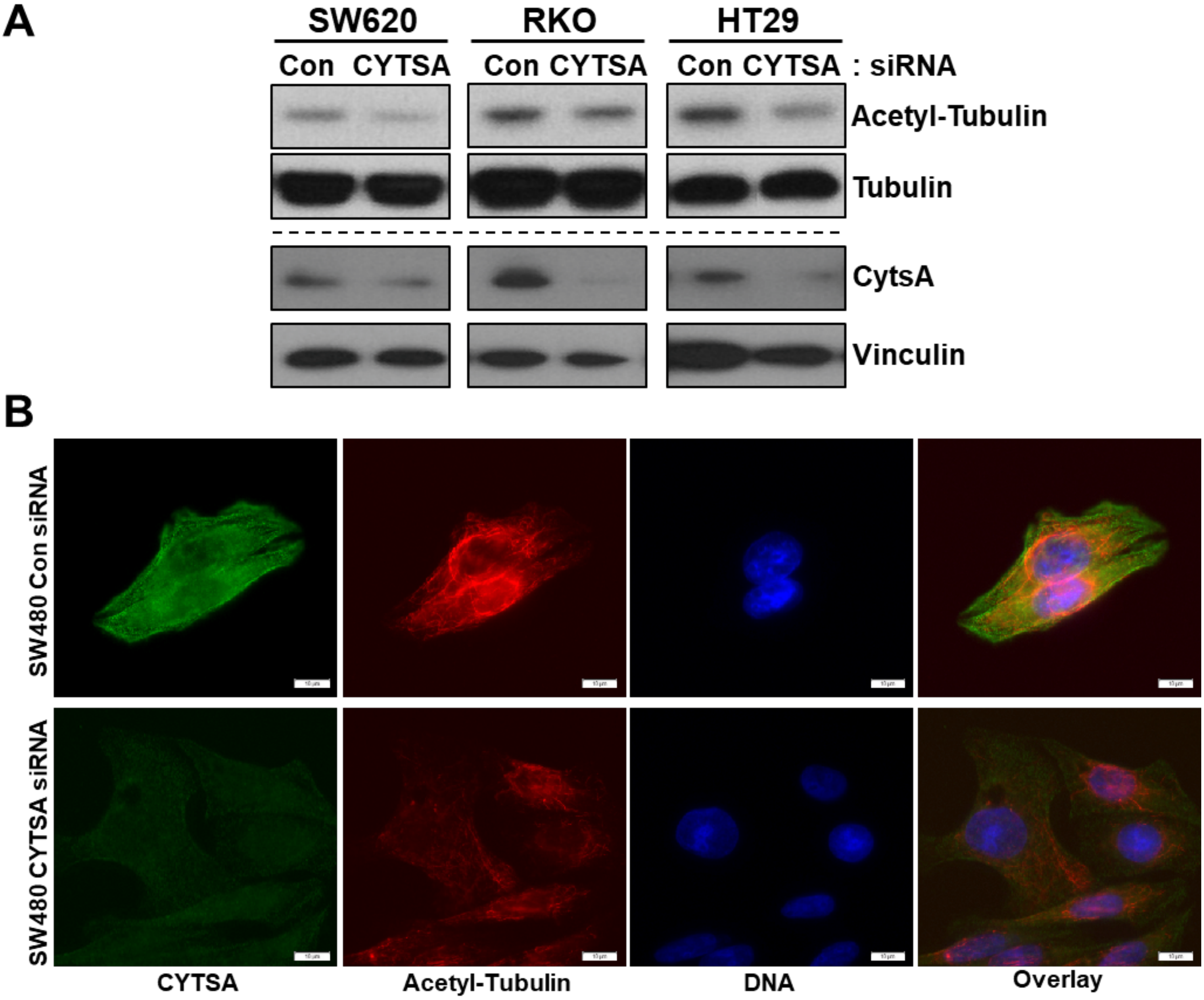

Supplementary Figure S5: CYTSA depletion reduces stability of microtubules in multiple CRC cells. A) Lysates of SW620, RKO and HT29 cells were analyzed for levels of acetylated α-tubulin and tubulin by western blotting. The same lysates were also analyzed for levels of CYTSA to validate knockdown of CYTSA. Vinculin was measured and loading control. **B)** SW480 cells transfected with either Con or CYTSA siRNA were fixed in methanol and stained with antibodies against CYTSA (green), acetylated α-tubulin (red). Nuclei were stained with Hoescht. Scale bar = 10 μm.

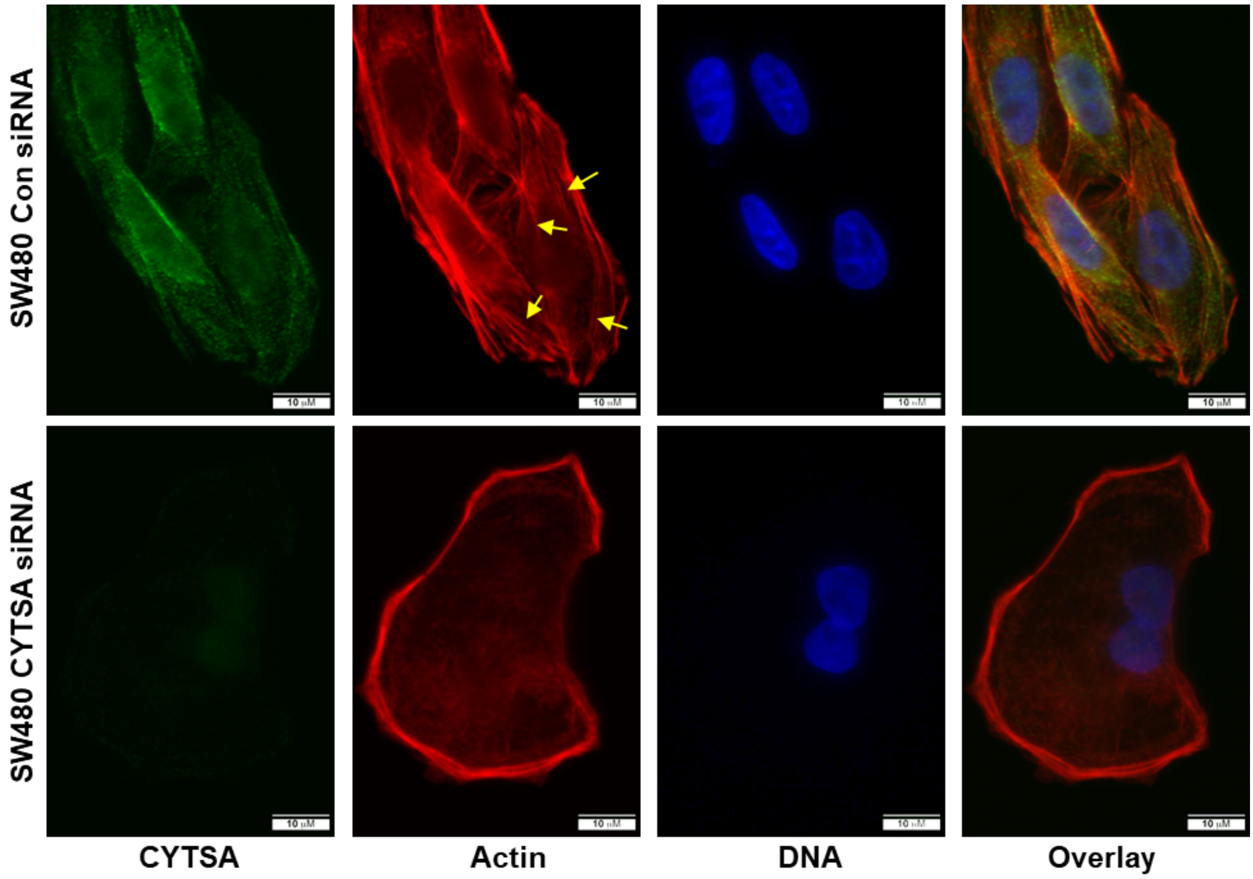

Supplementary Figure S6: Depletion of CYTSA alters actin polymerization in SW480 cells. SW480 cells were transfected with either Con siRNA or CYTSA siRNA and grown for 48 hours. Cells have been stained for CYTSA (green), actin (red) and DNA (blue). Con siRNA (top panels)- and CYTSA siRNA (bottom panels)-treated cells are shown. Scale bar = 10 µm.

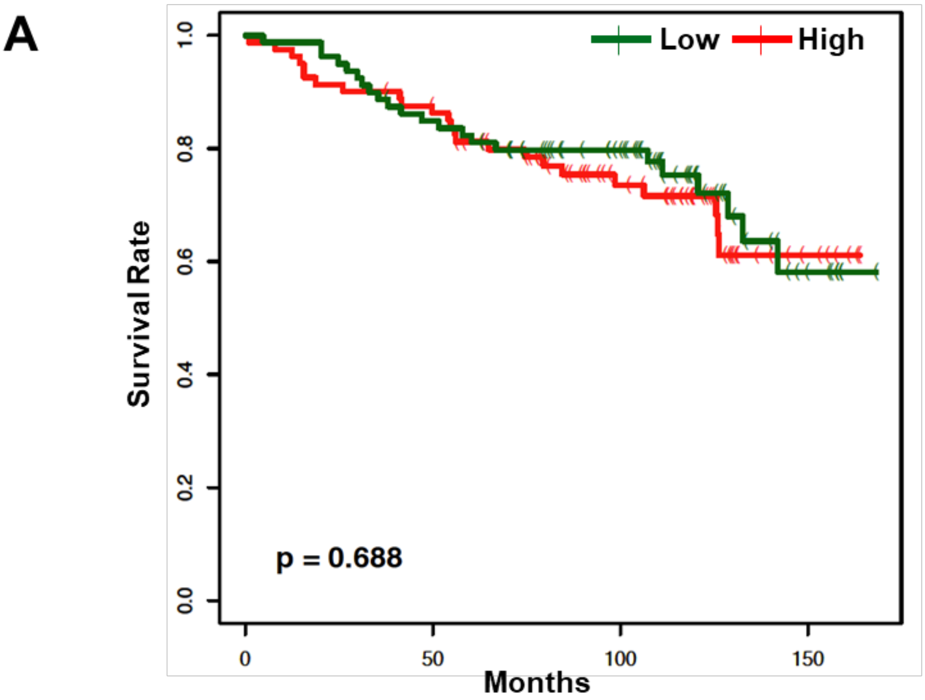

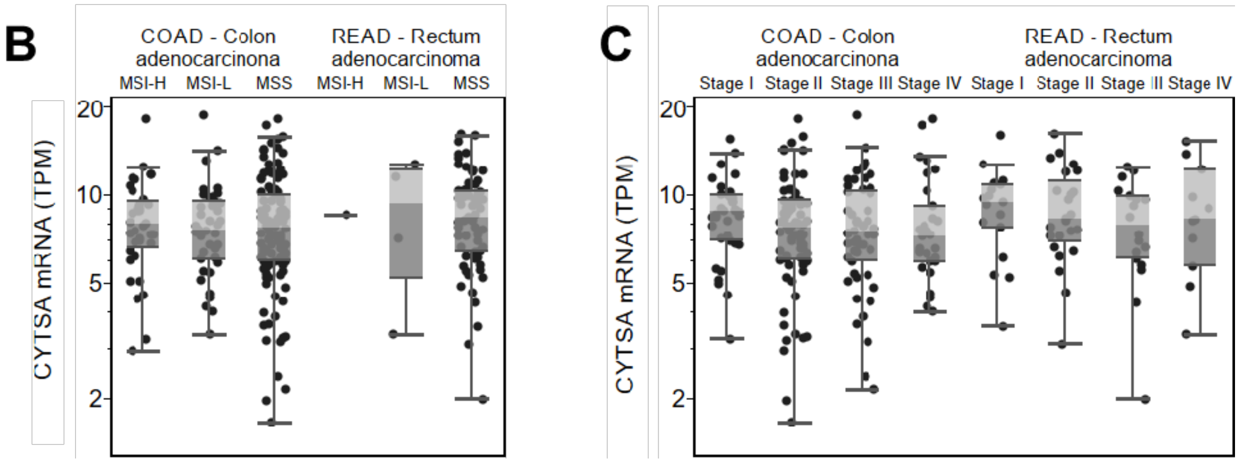

Supplementary Figure S7: Comparison of CYTSA expression levels and patient survival, and CRC tumors with different microsatellite instability status and stage. (A) Kaplan-Meier analysis of the MD Anderson Integromics colorectal adenocarcinoma data set showing survival of patients with lower CYTSA gene expression (n=79, green curve) vs high CYTSA expression group (n=80, red curve). P value is also shown. The TCGA colorectal cancer data set was analyzed for CYTSA expression with respect to microsatellite instability (B) and different stages of the disease (C). No significant changes in CYTSA expression in microsatellite instability high (MSI-H), microsatellite instability low (MSI-L) or microsatellite stable (MSS) were observed in either colon adenocarcinoma (COAD) or rectal adenocarcinoma (READ) samples (B). CYTSA expression was also not significantly different in COAD or READ samples from various stages of the disease (C).

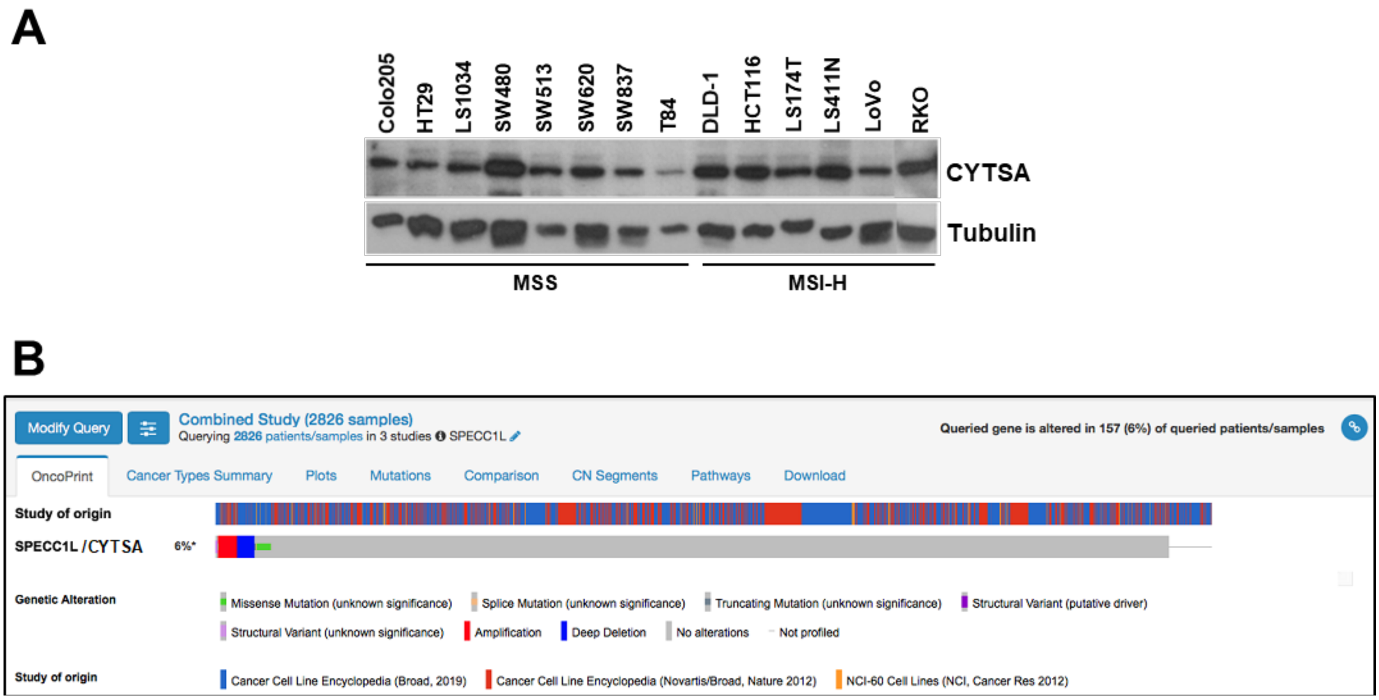

Supplementary Figure S8: CYTSA is expressed in almost all cancer cell lines. A) Various CRC cell lines that are microsatellite stable (MSS) or microsatellite instability high (MSI-H) were analyzed for expression of CYTSA protein by western blotting. Tubulin was used as loading control. B) Oncoprint data for CYTSA mutational status of ~2800 cancer cell lines was generated using the cbioportal website. ~6% cell lines have alterations in CYTSA protein expression or amino acid sequence. The various alterations are color coded and designated in the list of genetic alterations and the studies of origin are shown. Note: CYTSA is denoted as SPECC1L in the figure as the accepted gene name for performing queries in the cbioportal website is SPECC1L and not CYTSA.
