# Supplementary material for: Cytospin-A Regulates Colorectal Cancer Cell Division and Migration by Modulating Stability of Microtubules and Actin Filaments
Source: Cancers (Basel). 2022 Apr 14;14(8):1977. doi: 10.3390/cancers14081977 (PMC9026928; doi:10.3390/cancers14081977)
Supplement: Supplementary file 1 [file cancers-14-01977-s001.zip › cancers-1646065-WB.pdf]

Original blots for Figure 1

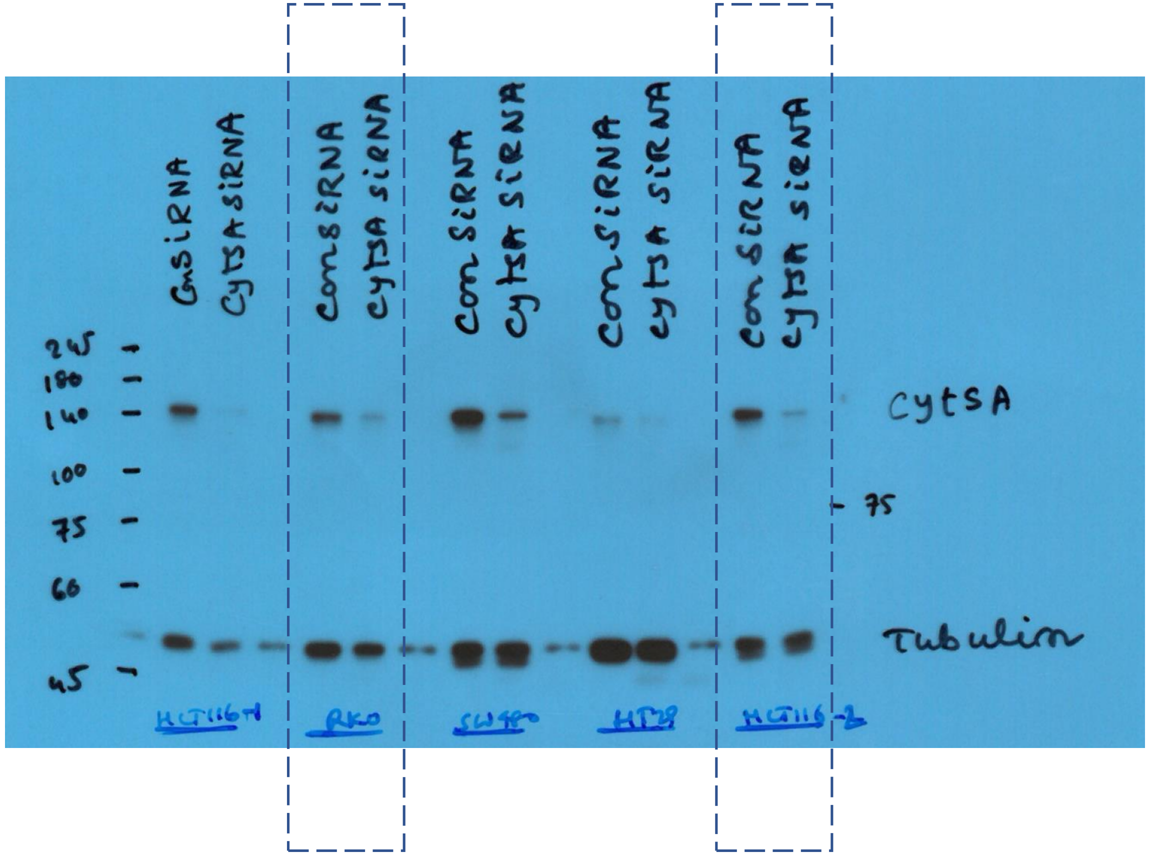

Original blots for Figure 5 and Supplementary Figure S4

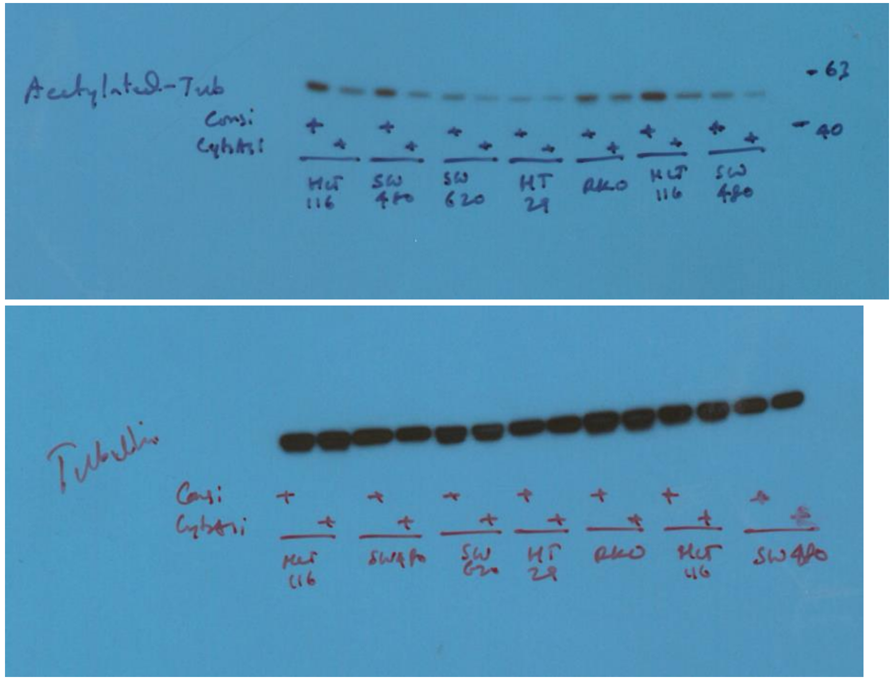

Acetylated tubulin

Tubulin

Acetylated tubulin and tubulin blots for Different CRC cells

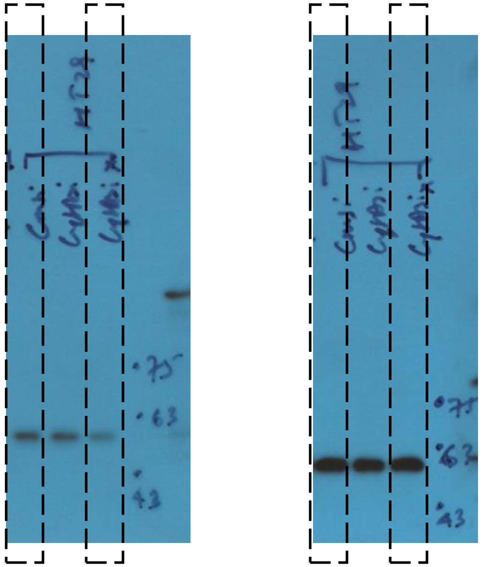

Acetylated tubulin and tubulin blots for HT29 cells

Original blots for Figure 5 and Supplementary Figure S4

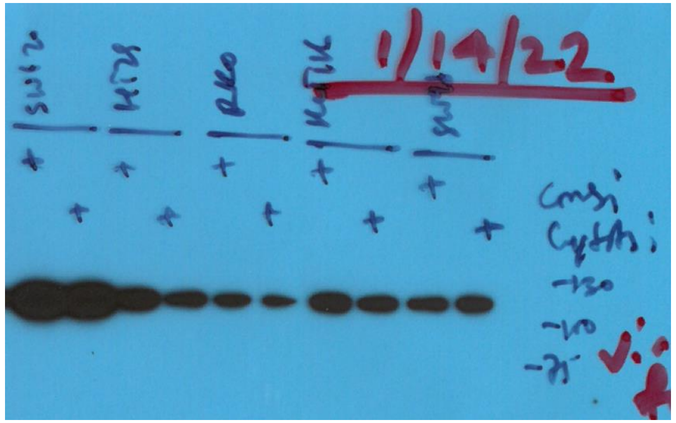

Vinculin
High exposure

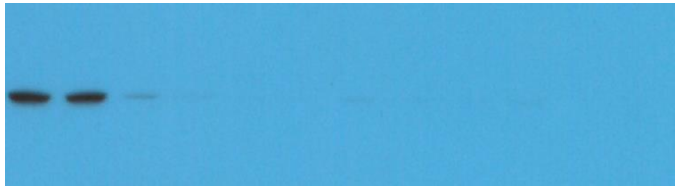

Vinculin
Low exposure

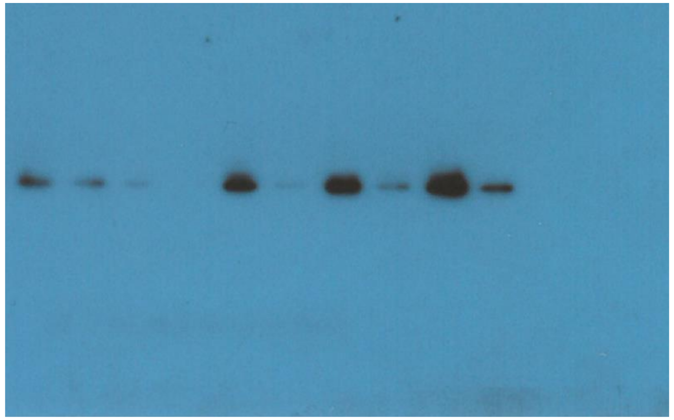

CytospinA
Low exposure

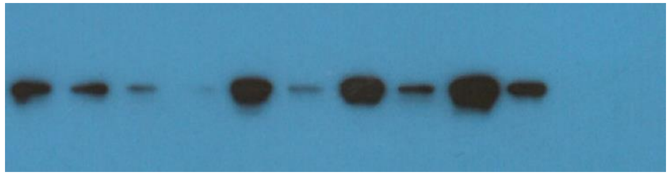

CytospinA
high exposure
